# Oral administration of Cystine and Theanine ameliorates oxaliplatin-induced chronic peripheral neuropathy in rodents

**DOI:** 10.1038/s41598-020-69674-9

**Published:** 2020-07-29

**Authors:** Takehiro Kawashiri, Daisuke Kobayashi, Nobuaki Egashira, Takashi Tsuchiya, Takao Shimazoe

**Affiliations:** 1 Department of Clinical Pharmacy and Pharmaceutical Care, Graduate School of Pharmaceutical Sciences, Kyushu University, Fukuoka, 812-8582 Japan; 2 Department of Pharmacy, Kyushu University Hospital, Fukuoka, 812-8582 Japan; 3 Department of Surgery, Sendai City Medical Center, Sendai City, Miyagi 983-0824 Japan

**Keywords:** Cancer models, Cancer therapy, Cancer, Neuroscience, Neurology, Oncology

## Abstract

Oxaliplatin frequently causes severe peripheral neuropathy as a dose-limiting toxicity. However, this toxicity lacks a strategy for prevention. Cystine/Theanine is a supplement, which includes precursors for the biosynthesis of glutathione. In this study, we investigated the effects of Cystine/Theanine on oxaliplatin-induced peripheral neuropathy using an in vivo model. Repeated injection of oxaliplatin (4 mg/kg intraperitoneally twice a week for 2 weeks) caused mechanical allodynia, cold hyperalgesia and axonal degeneration of the sciatic nerve in rats. Mechanical allodynia and axonal degeneration, but not cold hyperalgesia, were ameliorated by daily co-administration of Cystine [200 mg/kg orally (p.o.)] and Theanine (80 mg/kg p.o.). Moreover, co-administration of Cystine and Theanine to rats significantly increased the glutathione level in the sciatic nerve compared with the oxaliplatin group. Furthermore, Cystine and Theanine did not attenuate the tumour cytotoxicity of oxaliplatin in C-26 tumour cell-bearing mice. These findings suggest that Cystine and Theanine may be beneficial for preventing oxaliplatin-induced peripheral neuropathy.

## Introduction

Oxaliplatin, a platinum-based chemotherapeutic agent, is widely used for the standard treatment of colorectal, gastric, and pancreatic cancers, however frequently causes severe peripheral neuropathy. Acute neuropathy such as cold-related paraesthesia in hands, feet and the perioral region manifests within hours to days after oxaliplatin infusion. In almost cases, the cold-related acute neuropathy is transient and reversible^1,2^. Previous reports indicated that voltage-gated ion channels and transient receptor potential channels are involved in the acute neuropathy induced by oxaliplatin^3–5^. Sensory dysfunction as chronic neuropathy, which is a dose-limiting factor, occurs after repeated injections of oxaliplatin^2,6^. It is considered to be caused by morphological changes in neurons, such as axon degeneration and neuronal cell body damage^7–9^. However, no medications have been recommended to prevent chemotherapy-induced peripheral neuropathy^10^.

Cystine/Theanine, which is a supplement that contains l-Cystine (700 mg) and l-Theanine (280 mg), has been verified to affect adverse events, such as diarrhoea, oral mucositis and hand-foot syndrome in patients receiving cancer chemotherapy and during recovery after surgery^11–14^. Theanine is metabolised to glutamic acid and glutathione, which has an antioxidative effect, is synthesised from cysteine and glutamic acid. Many studies have indicated that oxidative stress plays a role in oxaliplatin-related peripheral neuropathy^15–17^. Furthermore, glutathione has reduced oxaliplatin-induced peripheral neurotoxicity in clinical studies^18,19^.

Therefore, we investigated the neuroprotective efficacy of Cystine/Theanine on oxaliplatin-induced peripheral neuropathy in rodent models.

## Results

### Effect of Cystine/Theanine on mechanical allodynia induced by oxaliplatin in rats

**Figure 1:**
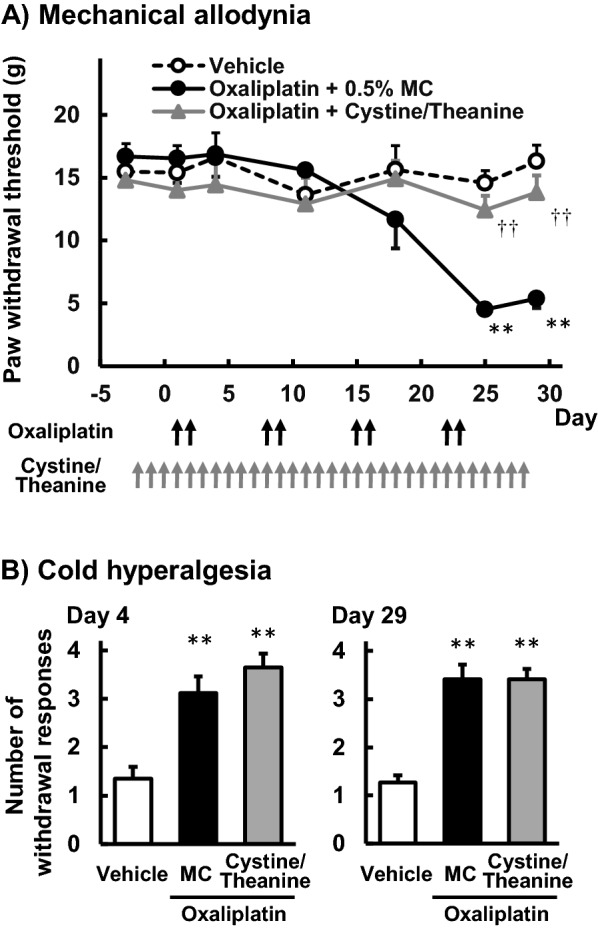
Effects of Cystine/Theanine on mechanical allodynia and cold hyperalgesia induced by oxaliplatin in rats. Oxaliplatin (4 mg/kg) was injected intraperitoneally twice a week for 4 weeks. Cystine/Theanine (200 and 80 mg/kg, respectively) was administered orally every day for 4 weeks. The von Frey test was performed before the first drug administration (day -2) and on days 4, 11, 18, 25, and 29 to assess mechanical allodynia (**A**). The acetone test was performed on days 4 and 29 to assess cold hyperalgesia (**B**). Data are expressed as the mean ± standard error of the mean (SEM). (n = 10). ^††^*P* < 0.01 compared with the vehicle; **P < 0.01 compared with oxaliplatin + 5% methyl cellulose (MC).

On the von Frey test, repeated administration of oxaliplatin (4 mg/kg i.p.) significantly reduced the paw withdrawal threshold compared with those with the vehicle treatment on days 25 and 29 (*P* < 0.01; Fig. 1A). Co-treatment with Cystine (200 mg/kg p.o.) and Theanine (80 mg/kg p.o.) significantly improved the reduction in the thresholds caused by oxaliplatin on both days (*P* < 0.01; Fig. 1A).

### Effect of Cystine/Theanine on cold hyperalgesia induced by oxaliplatin in rats

On the acetone test, the administration of oxaliplatin significantly increased the number of withdrawal responses to cold stimulus compared with those in the vehicle group on days 4 and 29 (*P* < 0.01; Fig. 1B). Co-treatment with Cystine and Theanine did not improve the increase in the number of withdrawal responses caused by oxaliplatin on both days.

### Effect of Cystine/Theanine on axonal degeneration of sciatic nerves induced by oxaliplatin in rats

**Figure 2:**
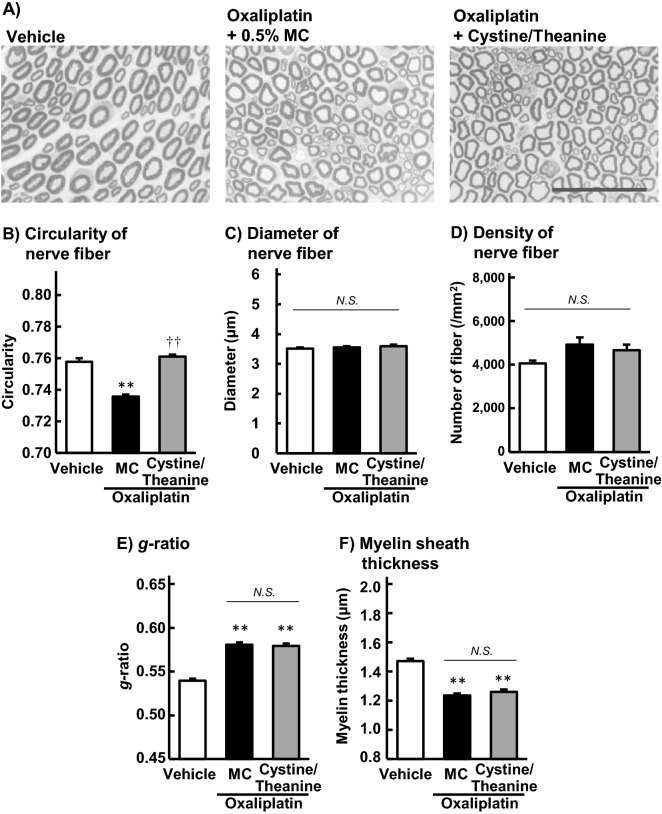
Effects of Cystine/Theanine on sciatic nerve morphological changes caused by oxaliplatin in rats. Oxaliplatin (4 mg/kg) was injected intraperitoneally twice a week for 4 weeks. Cystine/Theanine (200 and 80 mg/kg, respectively) was administered orally every day for 4 weeks. Sciatic nerves were harvested and stained with toluidine blue on day 30. The images (**A**) are magnified 40 × (bar = 50 µm). The circularity (**B**), diameter (**C**), and density (**D**) of nerve fibres, *g*-ratio (**E**), and myelin sheath thickness (**F**) were analysed using Image J 1.51 software. Data are expressed as the mean ± standard error of the mean (SEM). (n = 3). ^††^*P* < 0.01 compared with the vehicle; **P < 0.01 compared with oxaliplatin + 5% methyl cellulose (MC).

The axon circularities in the toluidine blue-stained sections of rat sciatic nerves significantly decreased in oxaliplatin group compared to the vehicle group on day 30 (*P* < 0.01; Fig. 2B). Co-administration of Cystine and Theanine significantly alleviated oxaliplatin-induced axon circularity reduction (*P* < 0.01; Fig. 2B). Neither the diameter of nerve fibres nor the density of fibres was changed by the administration of oxaliplatin (Fig. 2C and D). Although oxaliplatin induced an increase in the *g*-ratio, the ratio of the fibre diameter to the axonal diameter, and a decrease in the myelin sheath thickness (*P* < 0.01), co-administration of Cystine and Theanine did not change the *g*-ratio or the myelin thickness compared to the oxaliplatin group (Fig. 2E and F).

### Effect of Cystine/Theanine on the glutathione level in rat sciatic nerves

**Figure 3:**
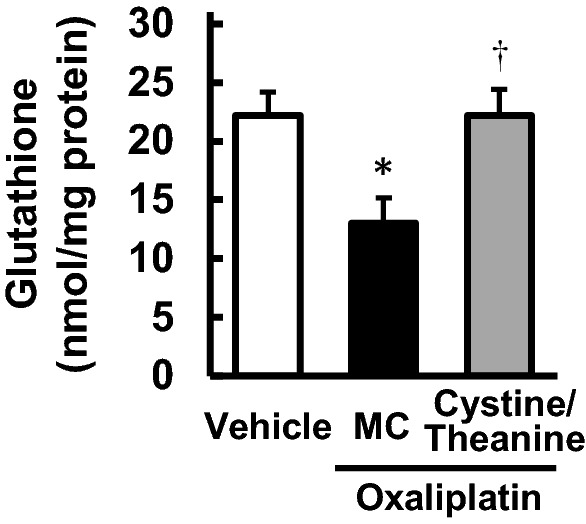
Effect of Cystine/Theanine on the glutathione level in rat sciatic nerves. Oxaliplatin (4 mg/kg) was injected intraperitoneally twice a week for 4 weeks. Cystine/Theanine (200 and 80 mg/kg, respectively) was administered orally every day for 4 weeks. Sciatic nerves were harvested, and the total glutathione content in tissues was measured on day 30. Data are expressed as the mean ± standard error of the mean (SEM). (n = 6). ^†^*P* < 0.05 compared with the vehicle; *P < 0.05 compared with oxaliplatin + 5% methyl cellulose (MC).

Repeated administration of oxaliplatin decreased the total glutathione content in the sciatic nerve on day 30 (*P* < 0.05; Fig. 3). Co-administration of Cystine and Theanine significantly reversed the reduction in the glutathione level compared with the oxaliplatin group (*P* < 0.05; Fig. 3).

### Effect of Cystine/Theanine on the antitumor activity of oxaliplatin in tumour cell-bearing mice

**Figure 4:**
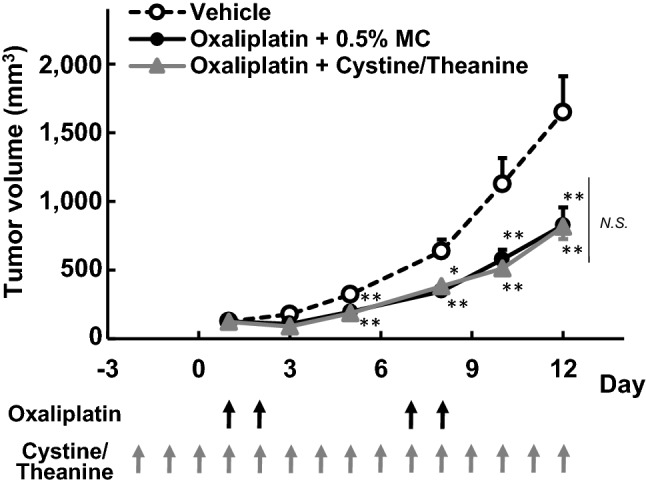
Effects of Cystine/Theanine on the antitumor activity of oxaliplatin. C-26 cell-bearing mice were treated with oxaliplatin (6 mg/kg intraperitoneally) twice a week and Cystine/Theanine (300 and 120 mg/kg/day, respectively; total 420 mg/kg/day; 210 mg/kg twice a day orally) every day for 2 weeks. The tumour volumes were calculated as follows: volume (mm^3^) = π/6 × thickness (mm) × length (mm) × width (mm). The tumour volumes are expressed as the mean ± standard error of the mean (SEM) (n = 10). *P < 0.05 and **P < 0.01 compared with the vehicle.

Repeated administration of oxaliplatin treatment (6 mg/kg i.p.) significantly attenuated tumour growth compared to the vehicle (*P* < 0.01; Fig. 4). Co-treatment with Cystine and Theanine (420 mg/kg/day; Cystine 300 mg/kg/day, Theanine 120 mg/kg/day; p.o.) had no effect in the inhibitory effect of oxaliplatin (Fig. 4).

## Discussion

Cystine/Theanine attenuated oxaliplatin-induced mechanical allodynia in rats, which mimics chronic neuropathy in a clinical setting. However, these amino acids did not reverse cold hyperalgesia, which was used to simulate oxaliplatin-related acute neuropathy, in this study. A previous study indicated there is a distinct difference between metabolites responsible for acute and chronic neuropathy caused by oxaliplatin; oxalate and platinum metabolites are involved in cold hyperalgesia and mechanical allodynia, respectively^20^. Other studies have reported that voltage-gated ion channels and transient receptor potential channels are involved in oxaliplatin-induced acute neurotoxicity^3–5^. However, it is thought that chronic neuropathy is caused by morphological changes in neurons, such as axon degeneration, neuronal cell body damage and demyelinating disease^7–9^. Our morphological experiment showed that Cystine/Theanine reversed the decrease in the circularity of nerve fibres but not the abnormalities in the *g*-ratio and myelin sheath thickness. It has been confirmed that the morphological changes are parallel to the behavioural changes in previous studies using oxaliplatin-induced peripheral neuropathy model^21–26^. The averages of circularities were 0.758 and 0.736 in vehicle and oxaliplatin groups, respectively. The difference, 0.022, was seemingly a mild change. However, it was a decent difference in comparison with the changes of the circularities in previous reports used diabetic neuropathy models, whose differences of the circularity between control and diabetic rats were 0.023–0.048, and oxaliplatin-induced neuropathy models, whose differences between control and neuropathy rats were 0.015–0.041^24–28^. Hence, it is thought that Cystine/Theanine ameliorates chronic neuropathic symptoms via protective effects against axon disorders (axonopathy) but not myelin abnormalities (myelinopathy).

Many previous basic studies support that oxidative stress plays a role in oxaliplatin-related peripheral neuropathy^15–17^. We have also shown the neuroprotective effects of some drugs, which had antioxidative activities, on oxaliplatin-induced peripheral neuropathy in rats and cultured cell lines^24,25,29^. Moreover, glutathione, which has an antioxidative effect, has reduced oxaliplatin-induced peripheral neurotoxicity in clinical studies^18,19^. Furthermore, oral administration of Cystine/Theanine increased the glutathione level in sciatic nerves in this study. Kurihara S and colleagues indicated that treatment of Cystine/Theanine increases the glutathione level in mouse liver^30^. Cystine and theanine are metabolized in the body to cysteine and glutamate, respectively. It is thought that glutathione is synthesized from cysteine and glutamate in monocytes, macrophages and dendritic cells^31^. Collectively, the increase in biosynthesis of glutathione from Cystine and Theanine might play an important role in the neuroprotective effect against oxaliplatin-induced neuropathy. Some reports have also indicated the possibility that glutathione conjugates to metabolites of oxaliplatin and inactivates platinum metabolites in cells^32,33^. Therefore, not only antioxidative activity but also conjugation to platinum might be involved in the amelioration of oxaliplatin-related neurotoxicity. Furthermore, some studies have reported that cystine and theanine reverse the increases in inflammatory cytokines, such as interleukin-6 (IL-6) and tumour necrosis factor α (TNF-α), in the mouse surgical model and the gut ischemia–reperfusion model^34,35^. The increases in IL-6 and TNF-α have reported in also cell and animal models of oxaliplatin-induced neurotoxicity^36,37^. It is also possible that the down-regulation of such inflammatory cytokines is partly involved in the neuroprotective effects of Cystine and Theanine.

Our study focused on the effects of oxaliplatin and Cystine/Theanine on the peripheral nerve system. However, it has been reported that oxaliplatin enhances the oxidative stress in also the central nerve system, such as brain and spinal cord^38–43^. Theanine can cross the blood–brain barrier (BBB) via L-type amino acid transporter 1 (LAT1) and LAT2^44,45^. Cystine is taken up by BBB endothelial cells via the complex of the cystine/glutamic acid transporter (xCT) and the heavy chain of 4F2 cell surface antigen (4F2hc) subunits^46,47^. It is thought that cysteine which decomposed from cystine in endothelial cells is transported into the brain via LAT1^48,49^. Taken together, it may be possible that systemic administration of Cystine/Theanine increases GSH in also the spinal cord and brain, and as a result, the neuropathy is suppressed via antioxidant action in the central nervous system.

The influence of Cystine/Theanine on anticancer activity of oxaliplatin was assessed in this study, and no impedance was observed in the tumour cell-bearing mice. Hence, Cystine/Theanine is unlikely to weaken the antineoplastic efficacy of oxaliplatin.

“Coasting” which means that the peripheral neuropathy continues to worsen for a time period despite cessation of therapy has been reported in patients treated with oxaliplatin^50^. In also our animal model, the mechanical allodynia persists approximately 20 days after the final administration of oxaliplatin^23,51^. However, the behavioural tests were assessed only until day 29 in this study. It is important to perform additional studies whether the neuroprotective effects of Cystine/Theanine is sustainable in the longer term. Moreover, further studies such as biochemistry and conduction velocity are also needed.

The present study demonstrates that repeated administration of Cystine/Theanine ameliorated oxaliplatin-induced mechanical allodynia and axon disorders without inhibiting antitumor efficacy in rodents. Therefore, Cystine/Theanine may be a preventive option for oxaliplatin-induced peripheral neuropathy.

## Methods

### Animals

Six-week-old male Sprague–Dawley rats weighing 200–250 g (Japan SLC, Inc., Shizuoka, Japan) were used for the oxaliplatin-induced peripheral neuropathy model. Six-week-old male BALB/c mice weighing 15–25 g (Japan SLC, Inc.) were used for the in vivo tumour growth model. Animals were housed in groups of 4–5 per cage with lights on from 7:00 to 19:00. Animals had free access to food and water in their home cages. All experiments were approved by the Experimental Animal Care and Use Committee of Kyushu University according to the National Institutes of Health guidelines and followed the International Association for the Study of Pain Committee for Research and Ethical Issues guidelines for animal research^52^.

### Drugs

Oxaliplatin (Elplat) was obtained from Yakult Honsha Co., Ltd. (Tokyo, Japan). Cystine/Theanine was a generous obtained from Ajinomoto Co., Inc. (Tokyo, Japan). In the oxaliplatin-induced peripheral neuropathy model, oxaliplatin (4 mg/kg) or a vehicle (5% glucose solution) was injected intraperitoneally (i.p.) twice a week for 4 weeks (days 1, 2, 8, 9, 15, 16, 22 and 23). Cystine/Theanine (Cystine 200 mg/kg and Theanine 80 mg/kg) or a vehicle (5% methyl cellulose) was administered orally (p.o.) every day for 4 weeks (days -2 to 28). Each drug was administered at a volume of 1 mL/kg. The doses of these drugs were determined based on previous reports^21,24,34^. Ten rats per group were employed for the von Frey test and the acetone tests.

### Von Frey test for mechanical allodynia

The von Frey test was used to evaluate the effect of Cystine/Theanine on mechanical allodynia. The test was performed before the first drug administration (day -2) and on days 4, 11, 18, 25 and 29 before drug administration. Each rat was placed in a wire mesh box for 30 min for adaptation before the test. Von Frey filaments (Aesthesio; DanMic Global LLC., San Jose, CA, USA) were applied to the mid-plantar skin of each hind paw for 6 s, and the paw withdrawal thresholds were determined using a modified up-down method as previously described^51^.

### Acetone test for cold hyperalgesia

The acetone test was performed to assess cold hyperalgesia. The test was performed only twice on days 4 and 29 because too many tests were distressing to the animals. The method was described in a previous report^20^. Rats were adapted to the wire mesh box in the same manner as that in the von Frey test. Acetone (FUJIFILM Wako Pure Chemical Corporation, Osaka, Japan) was sprayed onto the plantar skin, and the number of withdrawal responses was counted for 40 s.

### Assessment of sciatic nerve axonal degeneration

Sciatic nerves were harvested from three rats per group that were anaesthetised with sevoflurane (FUJIFILM Wako Pure Chemical Corporation) on day 30. After fixation of the samples in 2% (w/v) glutaraldehyde followed by 8% (w/v) sucrose substitution, nerves were embedded in Epon. Samples were sliced and stained with toluidine blue^51^. Sample sections were evaluated with light microscopy (BX51,Olympus, Tokyo) and analysed using ImageJ software version 1.51 (Wayne Rasband, National Institutes of Health, Bethesda, MD, USA). The circularity, diameter, *g*-ratio and myelin sheath thickness were acquired from 10,758 to 12,636 fibres of three animals per group. The circularity, the density of the nerve fibres, and the *g*-ratio were calculated by the Eqs. (1), (2) and (3), respectively.

1 $$ \left( {{\text{Circularity}}} \right) = \frac{{4 \times \uppi \times ({\text{axonal}}\;{\text{area}})}}{{\left( {{\text{Axonal}}\;{\text{perimeter}}} \right)^{2} }} $$

2 $$ ({\text{Density}}\;{\text{of}}\;{\text{nerve}}\;{\text{fibres}}) = \frac{{({\text{Number}}\;{\text{of}}\;{\text{axonal}}\;{\text{fibres}})}}{{({\text{Sciatic}}\;{\text{nerve}}\;{\text{area}})}} $$

3 $$ (g{\text{ } - \text{ Ratio}}) = \frac{{\left[ {({\text{Maximum}} \;{\text{of}}\;{\text{fibre}}\;{\text{ diameter}}) + ({\text{minimum }}\;{\text{of}}\;{\text{fibre}}\;{\text{diameter}})} \right] / 2}}{{\left[ {({\text{Maximum}} \;{\text{of}}\; {\text{axonal}} \;{\text{diameter}}) + ({\text{minimum}}\;{\text{ of}}\;{\text{ axonal}}\;{\text{diameter}})} \right] / 2}} $$

### Measurement of the glutathione level in sciatic nerves

Sciatic nerves were harvested from rats on day 30. The tissues were sonicated in 1 mL of saline and centrifuged at 15,000 rpm for 10 min. The total glutathione content in the supernatants was measured with a GSSG/GSH Quantification Kit (Dojindo Laboratories, Kumamoto, Japan).

### Assessment of the tumour growth in tumour-bearing mice

Murine colon adenocarcinoma (C-26) cells were obtained from the Cell Resource Centre for Biomedical Research, Tohoku University (Miyagi, Japan). C-26 cells were grown in Roswell Park Memorial Institute 1640 medium containing 2 mM l-glutamine and 10% foetal bovine serum. The cells were cultured in humidified air supplemented with 5% CO_2_ at 37 °C. C-26 cells (1.5 × 10^6^ cells) were subcutaneously implanted in the right paws of BALB/c mice. Three days after the implantation of the tumour cells, drug administration began. Oxaliplatin (6 mg/kg i.p.) was administered on days 1, 2, 8 and 9 and Cystine/Theanine (420 mg/kg/day; 210 mg/kg twice daily p.o.) was administered every day on days -2 to 12. The dose of oxaliplatin was determined based on previous reports^21,25,27^. Ten tumour-bearing mice per group were employed. The tumour volumes were measured on days 1, 3, 5, 8, 10 and 12 and were calculated as follows: volume (mm^3^) = π/6 × thickness (mm) × length (mm) × width (mm).

### Statistical analysis

The results are expressed as the mean ± standard error of the mean. Statistical analyses were performed using a one-way analysis of variance followed by the Tukey–Kramer test (Statview; Abacus Concepts, Berkeley, CA, USA) to determine the differences between the groups. A probability level of *P* < 0.05 was accepted as statistically significant.

## Data Availability

The data that support the findings of this study are available from the corresponding author upon reasonable request.
